# Rapidly Expanding Odontogenic Myxoma: An Entity With Diagnostic Challenges—A Case Report

**DOI:** 10.1155/crid/5741422

**Published:** 2025-04-14

**Authors:** Suwarna Dangore-Khasbage, Rajanikanth Kambala, Hanadi Sabban, Aakanksha Tiwari, Monika Khubchandani

**Affiliations:** ^1^Oral Medicine & Radiology, Sharad Pawar Dental College & Hospital, Datta Meghe Institute of Higher Education and Research, Deemed to be University, Wardha, Maharashtra, India; ^2^Oral and Maxillofacial Surgery, Sharad Pawar Dental College & Hospital, Datta Meghe Institute of Higher Education and Research, Deemed to be University, Wardha, Maharashtra, India; ^3^Oral Radiology Division, Oral Diagnostic Sciences Department, King Abdulaziz University Faculty of Dentistry, Jeddah, Makkah Province, Saudi Arabia; ^4^Oral Medicine & Radiology, Sharad Pawar Dental College & Hospital, Datta Meghe Institute of Higher Education and Research (DU), Wardha, Maharashtra, India; ^5^Pedodontics, Sharad Pawar Dental College & Hospital, Datta Meghe Institute of Higher Education and Research (DU), Wardha, Maharashtra, India

**Keywords:** clinical, histopathological, odontogenic, odontogenic myxoma, radiographic, radiolucent, surgical, tumor

## Abstract

Odontogenic myxoma is a rare benign odontogenic tumor having locally aggressive behavior. It frequently affects the females in the second or third decade of life and commonly occurs in the mandibular posterior region as a slow-growing lesion. The aggressive behavior is often seen in maxillary lesions. However, this case report describes odontogenic myxoma with aggressive or rapidly expanding behavior occupying the ramus and angle of the mandible in few months. An 18-year-old girl reported with a chief complaint of gradually increasing painless swelling in the posterior region of the mandible for 4 months. Extraoral examination revealed diffuse, firm to hard, nontender swelling on the left mandibular posterior region and intraorally missing mandibular left third molar with expansion in the buccal and lingual aspect in the mandibular posterior region. Radiographic features depicted large well-defined multilocular radiolucency and impacted mandibular left third molar. Based on clinical features and radiographic findings, ameloblastic fibroma, dentigerous cyst, unicystic ameloblastoma, and odontogenic keratocyst were the entities considered in differential diagnosis. However, the histopathological examination confirmed it as odontogenic myxoma which was then treated surgically. This concludes that though radiographic examination is the first step meant for diagnosis, odontogenic myxoma depicts variety of radiographic features mandating histopathological examination for confirmation of the diagnosis.

## 1. Introduction

Odontogenic myxoma (OM), being an odontogenic tumor, occurs in the tooth-bearing areas of the maxillary or mandibular jaw [1]. Among odontogenic tumors, its prevalence is 3%–6% with female predominance. They are usually asymptomatic. Nonetheless, pain, paresthesia, ulceration, and tooth mobility are the late-appearing symptoms [2].

As OM are the bony lesions, radiologic examination is the first option for diagnostic purpose. This includes conventional radiographs and advanced modalities which include computed tomography (CT), cone beam computed tomography (CBCT), and magnetic resonance imaging (MRI) [3, 4]. Irrespective of the maxilla or mandible, the radiographic features of OM are variable with reference to its periphery and internal structure as well as effect on surrounding structures that poses challenge in diagnosing the condition radiographically [3]. Nevertheless, histopathological examination is an indispensable tool for confirming the diagnosis which shows loosely arranged myxoid cells, fibroblasts in a myxomatous matrix, and the absence of a capsule. Calcification may or may not be present [5].

The management is surgical, and depending on certain factors like size, site, and age of the patient, surgical approach can be conservative to aggressive resections [6]. High recurrence rate is reported in the literature, and the justification for the same is absence of encapsulation in OM [1]. In contrast to the usual benign slow-growing behavior of OM, this article describes OM in a young female which increased aggressively and involved fairly a large portion of the mandible in a short duration of 4 months and this highlights the importance of this case.

## 2. Case Presentation

**Figure 1:**
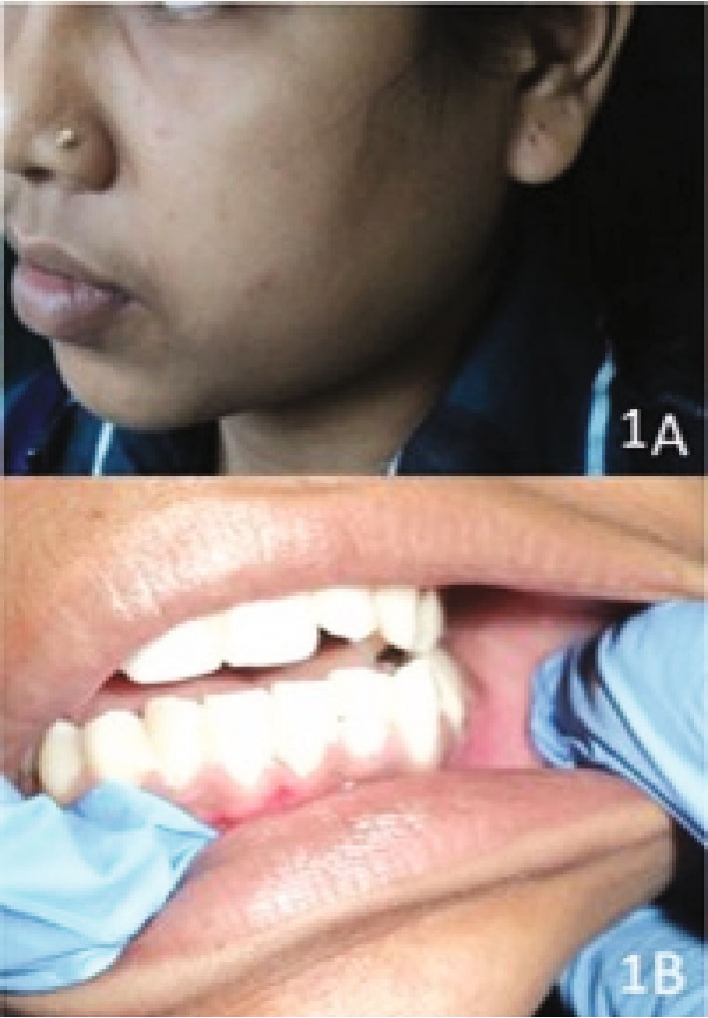
(A) Diffuse extraoral swelling in the left mandibular body. (B) Mild expansion in the posterior buccal self-area in 36–38 regions.

An 18-year-old female patient reported to the oral medicine and radiology department with a chief complaint of gradually increasing painless swelling in the posterior region of the mandible on the left side for 4 months. There was no history of mobility of the teeth or paresthesia in that region. Her medical history revealed that she was a physically and mentally healthy child since birth. There was no history of any blood disorder or blood transfusion in the past. Also, there was no history of trauma to the left side of the face since childhood. On extraoral examination, the face was asymmetrical due to diffuse, firm to hard, nontender swelling on the left mandibular posterior region, extending anteroposteriorly from the corner of the mouth to the angle of the mandible and superoinferiorly from the left ala tragus line to the inferior border of the mandible approximately 4 × 5 cm in size (Figure 1A). The overlying skin was normal with no signs of inflammation.

**Figure 2:**
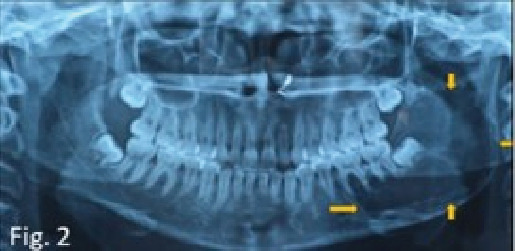
Orthopantomogram showing well-defined, multilocular, radiolucent lesion involving the angle and ramus of the mandible on the left side.

Intraoral examination revealed clinically missing mandibular left third molar, mild expansion in the buccal self-area in the mandibular posterior region, and mild expansion on the lingual side in the same region on palpation that was less than 1 × 1 cm on both buccal and lingual aspect (Figure 1B). No crepitus or crackling was present. No mobility, displacement, or tenderness of the teeth was present in the area of chief complaint. Based on clinical features, dentigerous cyst with developing mandibular left third molar, unicystic ameloblastoma, ameloblastic fibroma, and odontogenic keratocyst (OKC) were taken in differential diagnosis. An orthopantomograph was performed as the first investigation which showed developing all third molars, single large multilocular well-defined radiolucency associated with developing mandibular left third molar extending from the mesial aspect of the first molar to the posterior border of the ramus anteroposteriorly and from the sigmoid notch to the inferior border of the mandible in angle region superoinferiorly, causing expansion of the mandible, with thinning of the anterior, posterior, and inferior cortex (Figure 2). The internal density of the lesion is radiolucent with presence of internal septation, long and thin. The radiographic features were suggestive of benign behavior, which might be an odontogenic cyst or tumor.

**Figure 3:**
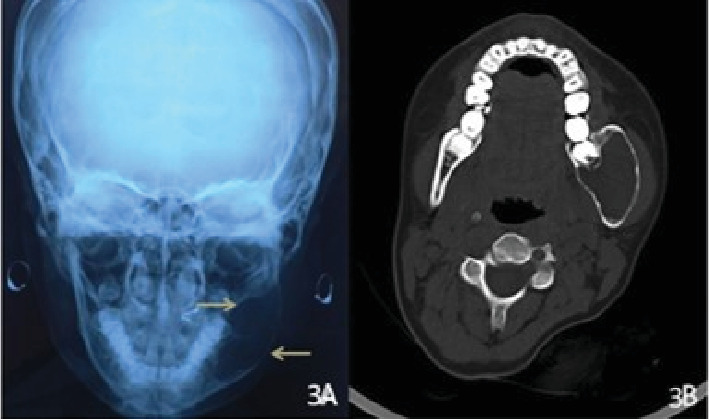
(A) Posteroanterior radiograph of the skull and (B) axial view of computed tomography showing expansion of the buccal and lingual cortical plates with thinning.

In addition to this, to appreciate the buccal and lingual expansion, a posteroanterior radiograph of the skull was performed that revealed expansion of the buccal and lingual cortical plates with marked thinning of the buccal cortex as shown in Figure 3A.

Considering the limitations of conventional imaging like superimposition of the structures, inability to depict the exact extension of lesion, and the effect on surrounding structures, the lesion was further evaluated by performing CT. CT (axial view) showed a large hypodense and well-defined lesion involving the posterior part of the body of the mandible and ramus of the mandible with marked expansion of the buccal and lingual cortical plates with thinning in the same region. But no perforation of the cortical bones was present (Figure 3B). The diagnosis was consistent with the diagnosis of conventional imaging.

**Figure 4:**
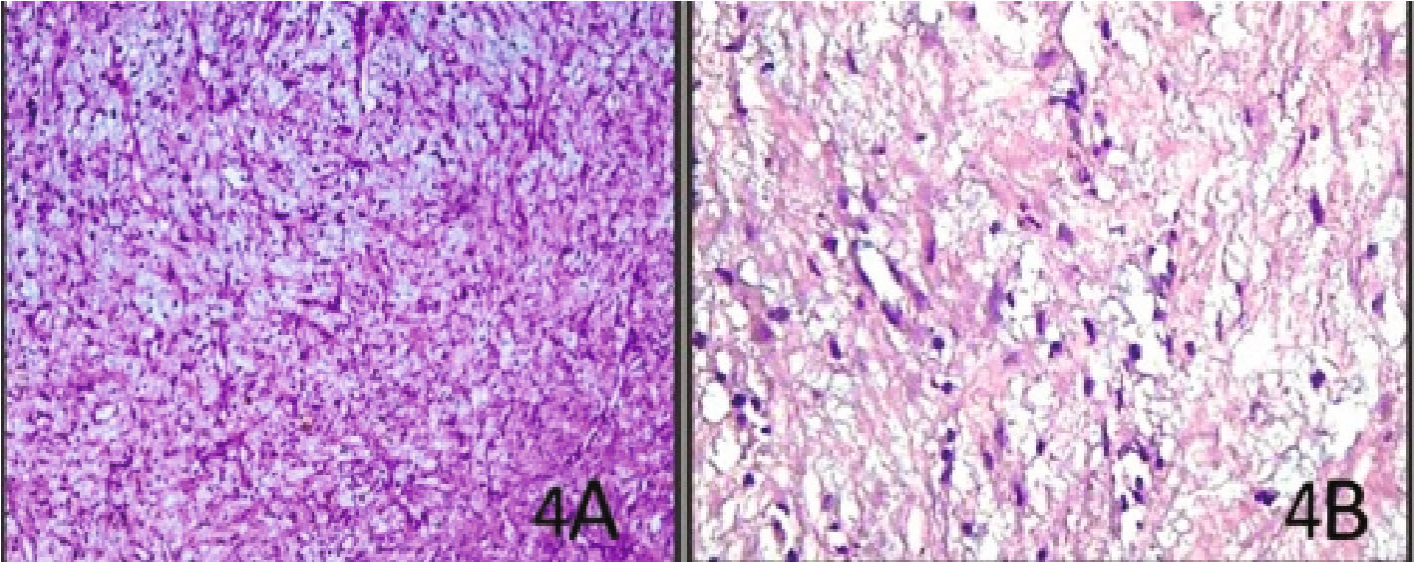
(A) Low-power photomicrograph illustrating loosely arranged myxoid cells with few collagen fibrils and capillaries (H and E, ×10). (B) High-power photomicrograph showing myxoid cells and plump-shaped fibroblasts (H and E, ×40).

Bearing in mind the multilocular appearance of the lesion, with the presence of internal septation, the radiographic diagnosis was more in favor of benign odontogenic tumors like ameloblastic fibroma, unicystic ameloblastoma, OM, and keratocystic odontogenic tumor. Biopsy was performed to confirm the diagnosis before surgery. The low-power view (10×) showed loosely arranged myxoid cells, and the high-power view showed myxoid cells and plump-shaped fibroblasts on hematoxylin (H) and (E) eosin staining, features suggestive of OM (Figure 4A,B).

**Figure 5:**
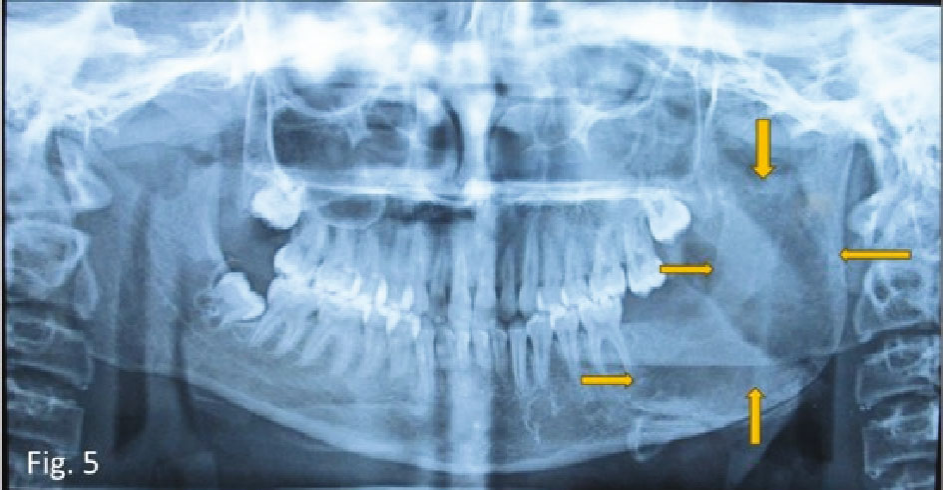
Postoperative orthopantomogram depicting well-defined radiolucency of the surgical area in the angle and ramus of the mandible on the left side.

The lesion was treated surgically by enucleation, curettage, and chemical cauterization of the bony cavity with extraction of the mandibular left second and third molars under general anesthesia. Chemical cauterization was done with Carnoy's solution for 3 min. Hemostasis was achieved, and wound closure was achieved with an absorbable suture. The patient was hospitalized for 5 days after surgery, and she was on intravenous antibiotics and analgesics for these 5 days. No complications were noted on subsequent follow-ups, rather a healthy healing being noted.  Figure 5 depicts single large radiolucency in the body and ramus of the mandible on the left side suggestive of surgical defect.

The surgical specimen was further evaluated by histopathological examination which confirmed OM. The regular follow-up of the patient is maintained for 12 months after surgery, and clinical examination revealed no evidence of recurrence till now. Nevertheless, strictly periodic follow-up will be undertaken to assess recurrence for 5 years.  Table 1 describes stepwise clinical and diagnostic events chronologically.

## 3. Discussion

OM is a rare lesion with a benign pathogenesis but aggressive behavior [2, 7]. In the head and neck, myxomas occur in bone and soft tissues. Myxoma in bone affects facial skeleton including the mandible and the maxilla. Those of soft tissue are identified within skeletal muscle, subcutaneous tissues, and fascial planes.

The possible sites of origin for OM are reported to be dental papilla, the follicle as OM thought to originate from the derivatives of neural crest ectomesenchyme. The similarity of histopathological features of OM with the dental papilla supports this pathogenesis. Nevertheless, occurrence of OM at nonodontogenic sites like in a condyle or in an edentulous jaw of elderly where the odontogenic apparatus is probably nonfunctional queries this pathogenesis [8, 9]. Thus, Naidu et al. [9] mentioned that the cause of jaw myxomas is still controversial.

The common age of occurrence of OM is from 10 to 40 years, with a peak incidence in the third decade of life. The present report describes OM in an 18-year-old female. Similar cases of occurrence of OM in younger age group are reported in the literature [2, 10, 11]. Although OM is common in females, Zhang et al. reported more prevalence in males [12]. The common location is the mandibular posterior region. There are cases with rare location such as in the mandibular anterior region, condyle, or maxilla which are also documented in the literature [8, 13, 14].

There are certain similarities between OM and unicystic ameloblastoma, ameloblastic fibroma, and OKC. All are common in young adults (second to third decades), ameloblastic fibroma peak in the first to second decades. All mostly affect the mandible that too in the posterior region. Radiographically, all have well-defined periphery, and as slow-growing lesions, all these benign tumors cause root resorption and displacement of adjacent structures and may cause bony expansion. All these can have pericoronal presentation. OM and OKC have a higher recurrence rate compared to ameloblastoma and ameloblastic fibroma, which tend to have lower recurrence rates if treated properly. These similarities and the comparison shown in Table 2 can assist the clinicians in differentiating these four entities from each other when evaluating clinical and radiographic features [15–20].

The diagnosis of OM is usually based on radiographic and histopathologic findings. Traditionally, the conventional radiographs are performed as an initial investigation and then followed by advanced imaging techniques. OM usually presents as a multilocular radiolucency with well-defined or diffused borders having varied appearances described as “soap bubble, honeycomb, or tennis racket.” Nevertheless, it may have unilocular radiolucent appearance too. Controversial reports are existing pertinent to its tendency to cross the midline and such OM are usually maxillary with multilocular presentation [21]. Regarding periphery, also variable findings are mentioned in the literature [3, 11, 21, 22]. The periphery of OM can be well defined or ill defined. If well defined, it can be corticated and noncorticated. Unilocular type usually has well-defined periphery. Bone perforation as well as periosteal reactions like “sun-ray” or “sun-burst” appearance may be found in larger sized ill-defined OM with multilocular presentation [23].

Zhang et al. [12] reported six types of radiographic appearances of OM as Type I—unilocular, Type II—multilocular (including honeycomb, soap bubble, and tennis racket patterns), Type III—involvement of local alveolar bone, Type IV—involvement of the maxillary sinus, Type V—osteolytic destruction, and Type VI—a mix of osteolytic destruction and osteogenesis [12]. In scanty cases, OM may be associated with unerupted tooth/teeth [24]. All these variable radiographic features represent difficulty in diagnosing OM based on only radiographic features.

The present case revealed large rapidly expanded multilocular radiolucency having well-defined and scalloped borders in the vicinity of impacted mandibular left third molar on OPG. Considering the close proximity of lesion with impacted third molar, additional radiographic examination was performed to rule out various pericoronal lesions such as ameloblastic fibroma and pericoronal variety of OM. Both posteroanterior view and CT scan showed that mandibular left third molar was just close to the radiolucent lesion, while OPG being a two-dimensional imaging method, it was superimposed over the radiolucency. These limitations of convention radiography warrant use of advanced imaging in OM.

Conventional radiography fails to show the growth pattern of the tumors thus unable to depict the exact extension of the lesion. The margins and cortication of OM may not be revealed precisely. Conventional radiography also lacks to differentiate between various components of the tumor. This explains the superiority of advanced imaging and may dictate the necessity of using these techniques in diagnosis and presurgical assessment of OM [3].

CT and MRI offer the advantage of determining the extension of the tumor in the different planes. This benefit greatly affects the management of maxillary tumors where there is extension of the tumor into the cranial base or floor of the orbit and paranasal sinuses. The high spatial resolution available on CT accounts for its ability to show resorption of the teeth, determining densities of the tumors. CT was found to show the extension of the tumors, status of cortication, expansion, locularity, and extension into the surrounding structures. Moreover, CT, by determining the attenuation values of the tumors, is able to compare the densities of the tumor with the surrounding muscles [3].

MRI shows the extension of the tumor, contents, and pattern of growth of the tumor. Additionally, MRI can differentiate between various soft tissues and determine any invasion of the tumor into adjacent soft tissues. The maxillary lesions have a tendency to encroach the maxillary sinus, while the mandibular OM surrounds the mandibular canal [25]. MRI is useful for differentiating the myxoma's density from surrounding soft tissues and for delineating lesion margins. Advanced sequences, such as diffusion-weighted imaging (DWI), can assess the internal content, reflecting the lesion's myxoid nature [3, 26]. CT and MRI greatly aid in diagnosing the OM and differentiating it from other tumors with similar presentation. Being a modality with a high spatial resolution, advanced imaging may facilitate the determination of the intraosseous extent of the tumor and guide the surgeon in the planning of the resection margins [26].

Similarly, CBCT signifies its utility in detailing the comprehensive internal structures of the lesions more critically. This includes arrangement of septa representing specific pattern. CBCT also provides information regarding margins, effect on alveolar process, and cortical alterations and assesses bone expansion [4]. Therefore, the use of advanced imaging in addition to conventional radiography should be routine in the diagnosis of OM because of its soft tissue–invading features.

A multilocular radiolucency is the commonest radiographic appearance of OM in which other bony lesions with multilocular variety such as ameloblastoma, aneurysmal bone cyst, central giant cell granuloma, OKC, and hemangioma should be well-thought-out in differential diagnosis. In unilocular radiolucent presentation, differential diagnosis includes dentigerous cyst, unicystic ameloblastoma, and ameloblastic fibroma. Overall, it seems that radiographic examination just provides clue regarding diagnosis and differential diagnosis.

Although OM is classified as a benign tumor, it possesses the potential for locally invasiveness into surrounding tissues, sometimes leading to extensive bone involvement. In such rapidly expanding osteolytic lesions, a biopsy is often performed initially to establish a diagnosis and planning an appropriate surgical strategy as executed in the present case. Nonetheless, in the small and slow-growing OM, the diagnosis is inconclusive based on clinical and radiographic features, which mandates histopathological confirmation. With reference to histopathological features of OM, Choudhary et al. [27] stated that the myxoma is bland in appearance and is composed of loosely arranged, evenly dispersed spindle-shaped, rounded, and stellate cells with a lightly eosinophilic cytoplasm in a mucoid-rich (myxoid), intercellular matrix. Many stellate tumor cells have anastomosing, long tapering, and cytoplasmic processes. Some degree of mild nuclear pleomorphism or hyperchromatism may exist, including an occasional mitosis or binucleate cell [27]. It is reported that although the immunohistochemical analysis may help in diagnosis, that plays no role in guiding treatment planning or predicting the rate of recurrence [27].

It is well known that the indispensable tool for confirming the diagnosis is histopathology. Histologically, OM shows a spectrum of fibrous connective tissue stroma, from myxoid to dense hyalinized and from relatively acellular to cellular. Calcification may or may not be present.

The choice of treatment for OM is surgical. Yet, a controversy exists regarding the surgical approach to be chosen in a specific case. Surgical approach can be conservative to aggressive. Conservative approach involves enucleation and curettage of the lesion with an intension to preserve the uninvolved structure to maintain function. On the contrary, the aggressive surgical approach refers to segmental resection and hemimandibulectomy [1, 2, 7, 28]. A number of factors which include age of patient, site and size of lesion, involvement of the maxilla or mandibula decide the type of surgical method to be used. Maxillary OM is traditionally treated by wide surgical excision as this is aggressive. Boffano et al. [29] recommended conservative approach for the OM smaller than 3-cm diameter though segmental resections are preferred in larger tumors.

Accordingly, the surgical resection would be the preferred treatment in the present case. Although considering the patient's age and potential complications associated with surgical resection, surgical enucleation and curettage of the lesion were done along with extraction of the mandibular left second and third molars under GA. Also, the chemical cauterization was done with Carnoy's solution in order to prevent recurrence. A similar treatment protocol is reported in the literature especially for OM in young patients [30, 31].

Enucleation and chemical cauterization refer to a surgical treatment for an OM where the tumor is carefully removed from its bony cavity (enucleation) and then the remaining bony walls are treated with a chemical agent (like Carnoy's solution) to reduce the risk of recurrence.

Routinely, chemical cauterization using Carnoy's solution is used as an adjuvant therapy in the management of OKC considering its high recurrence rate. However, Lal et al. [32] in their systematic review reported its application in a number of benign lesions including OM. They stated that the use of Carnoy's solution is not limited to treat OKC but it has also been extrapolated in the management of other even large benign lesions of the jaws with the intent to prevent a recurrence due to its property to penetrate the cancellous spaces and thus devitalization and fixation of the residual tumor cells [32, 33]. Nonetheless, the use of chemical cauterization remains a controversial topic as it may cause damage to the nerves and vessels, wound dehiscence, infection, bone sequestrum, etc., but the process is safe for exposure times less than 5 min. Its application should be better restricted to 3 min to avoid neural and vascular injury [34].

OM has high recurrence rate of approximately 25%. The multiple reasons for high recurrence rate are (i) myxomatous nature of the tumor, (ii) lack of a capsule, and (iii) propensity of tumor to penetrate the surrounding tissues. These points should be taken into consideration during surgical planning of a case of OM.

The study by Martins et al. [35] reported successful conservative approach in the follow-up of up to 10 years. They mentioned that enucleation combined with modified Carnoy's solution can be adopted as a first-line treatment modality to OM to prevent recurrence [35]. With refence to high recurrence rate, the resection of a tumor with a bony margin of 1 cm is also recommended in the literature [22]. The recurrence usually occurs within 2 years; nonetheless, follow-up is advised for 5 years.

## 4. Conclusion

The radiological features of OM are variable and are often comparable to quite a few other tumors, cysts, and reactive lesions of the jaws. However, careful interpretation of the radiographic features aids in providing the direction for accurate diagnosis. Though histopathological examination is a must to confirm the diagnosis, radiographic examination plays a vital role in diagnosis and differential diagnosis of OM.

## Figures and Tables

**Table 1:** Stepwise table describing the clinical and diagnostic events chronologically.

**Steps**	**Task performed**	**Diagnosis and differential diagnosis**	**Further plan**
Step 1	Thorough clinical evaluation	Benign lesion either odontogenic cyst or odontogenic tumor, for example, ameloblastic fibroma, unicystic ameloblastoma, dentigerous cyst with developing mandibular left third molar, odontogenic keratocyst (OKC), and odontoma	Radiographic evaluation
Step 2	Orthopantomograph Posteroanterior view of the skull	Benign odontogenic tumor ameloblastic fibroma, unicystic ameloblastoma, OM, and keratocystic odontogenic tumor	Advanced imaging—computed tomography
Step 3	Computed tomography	Benign odontogenic tumor like ameloblastic fibroma, unicystic ameloblastoma, OM, and keratocystic odontogenic tumor	Histopathological evaluation
Step 4	Biopsy	Characteristic features on H&E staining suggestive of OM	Surgical plan
Step 5	Surgical excision of the lesion	Enucleation, curettage, and chemical cauterization under GA	Postoperative evaluation of the surgical specimen and postoperative radiographic evaluation
Step 6	Follow-up	Histopathological features of surgical specimen were consistent with OM	Yearly periodic follow-up to assess recurrence for 5 years

**Table 2:** Comparison of clinical features, radiographic features, and recurrence rate of OM with similar conditions.

**Features**	**Odontogenic myxoma**	**Unicystic ameloblastoma**	**Ameloblastic fibroma**	**Odontogenic keratocyst**
Clinical features	Mostly in the mandible (posterior body/ramus area) Asymptomatic, may cause swelling, sometimes pain Soft, gelatinous, or spongy consistency on palpation Expansile, aggressive, may cause bone resorption and displacement [1, 15]	Mandible (most commonly) or maxilla May be asymptomatic or cause swelling and discomfort Firm or slightly soft depending on the stage Slow-growing, expansile, with a tendency to be locally invasive [16]	Mandible (mostly premolar and molar region) Swelling, painless, sometimes may present with pain Firm, similar to solid tumors Expansile, slow-growing, locally aggressive [17, 18]	Mostly mandible, especially posterior body/ramus area Asymptomatic, may present with swelling or pain Cystic, with a fluid-filled consistency Expansile, aggressive, can cause significant bone destruction [19, 20]

Radiographic features	Multilocular, “soap bubble” or “honeycomb” or “tennis racket” appearance, may have thin corticated borders Thinning and expansion of cortical bone, can perforate the cortical plate Displacement or resorption of adjacent tooth roots [11, 15, 21]	Unilocular radiolucency, well-defined borders, pericoronal Cortical expansion without perforation in many cases Minimal or no root resorption, often preserves [16, 22]	Unilocular or multilocular radiolucency with well-defined borders Cortical expansion, may involve adjacent structures May involve adjacent tooth roots, can cause resorption [18, 22]	Unilocular or multilocular radiolucency with well-defined borders Cortical expansion, rarely perforates the cortical plate Often displaces or resorbs tooth roots [20]

Recurrence rate	High [15]	Moderate with potential for recurrence if not treated adequately [16]	Low, rare recurrence when excised properly [17, 18]	High recurrence rate [20]

## Data Availability

The data that support the findings of this study are available from the corresponding author upon reasonable request.
